# Bites by Non-Native Reptiles in France: Species, Circumstances and Outcome

**DOI:** 10.3390/toxins14080570

**Published:** 2022-08-20

**Authors:** Gaël Le Roux, Guillaume Grenet, Corinne Schmitt, French Poison Control Centers Research Group, Sébastien Larréché, Alexis Descatha

**Affiliations:** 1 ‘Grand Ouest’ Poison Control Center, University Hospital of Angers, CEDEX 9, 49933 Angers, France; 2 Institut de Recherche en Santé, Environnement, Travail (IRSET, INSERM UMR_S 1085, ESTER Team), CEDEX 1, 49045 Angers, France; 3 Lyon Poison Control Center, HCL, 69003 Lyon, France; 4 Laboratory of Biometry and Evolutionary Biology UMR 5558, CNRS, University of Lyon 1, CEDEX, 69622 Villeurbanne, France; 5 ‘Sud’ Poison Control Center, APHM, 13005 Marseille, France; 6 Departement of Medical Biology, Bégin Military Teaching Hospital, 94160 Saint-Mandé, France; 7 INSERM UMR-S1144, University of Paris Cité, 75006 Paris, France

**Keywords:** exotic reptile, snakebite, poison control center, antivenom

## Abstract

We aimed to make an exhaustive assessment of circumstances of bites by exotic reptiles bred in France. A retrospective observational study was conducted in all the reported cases from 2000 to 2020 in French poison control centers (PCCs). Two hundred and eighteen cases of bites were recorded. The sex ratio (M/F) of the patients was 1.79 and the mean age of the patients was 29.0 ± 15.8 years. Twenty-two cases (10.1%) occurred during the deep night. One hundred and eighty-six bites (85.7%) occurred in a private context; however, there were more cases of high severity when it occurred in a professional setting (60.0% vs. 11.2%, *p* < 0.01). The feeding/nursing activity accounted for 54.7% cases. Forty-three species of snake were identified; 28 were considered venomous. There were no deaths among the patients in the study. Most of the cases (85.8%) were of mild severity. All of the patients bitten by a venomous reptile were hospitalized: 10 patients received an antivenom; and 2 required surgery. Bites occurred at home and by a small number of popular non-venomous reptile species (pythons and boas, colubrids). These occurred mainly when handling the animals. The rare envenomations were mainly by Asian and American crotalids, followed by elapids. One-third of them were treated with antivenom when available.

## 1. Introduction

Ophidian envenomations are uncommon but potentially serious in Western Europe. They affect 0.4 to 1.1 people per 100,000 inhabitants per year and cause approximately 4 deaths each year [1]. *Vipera sensu strictu* is the genus most associated with venomous snakebites. The most common species are: *V. berus*, which is also the species with the widest distribution in Europe; followed by *V. ammodytes*, *V. aspis*, *V. latastei*, *V. ursinii*, and *V. seoanei* [2].

Alongside these native species, many exotic reptiles are kept in professional or private breeding facilities. A recent study estimated that there are approximately 1250 species of snakes in international trade [3], including venomous animals.

The main objective of this study was to describe frequency of bites by exotic reptiles in France, in terms of the species involved and management.

## 2. Results

**Figure 1:**
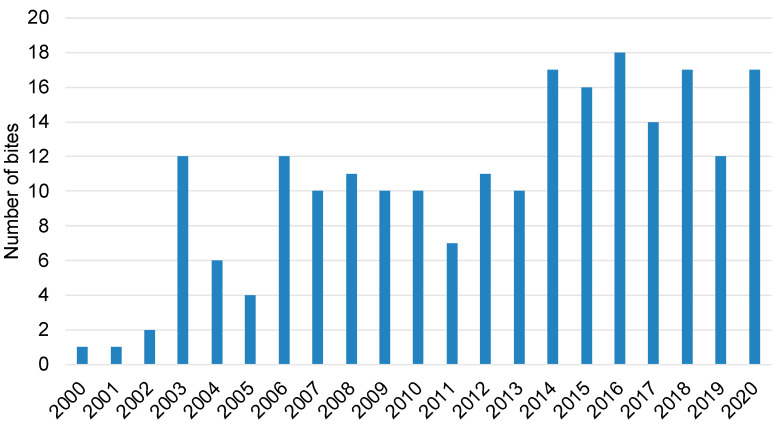
Number of annual cases of bites by an exotic reptile.

**Figure 2:**
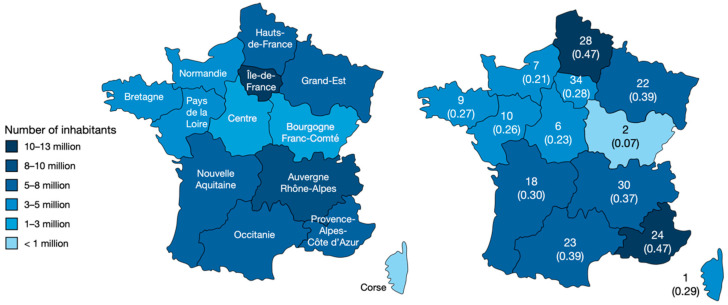
Number and incidence of non-native reptile bites in metropolitan France. On the left, the French regions with the number of inhabitants; the variation in color corresponds to the population. On the right, the number of cases (and incidence/100,000 inhabitants) for each region; the variation in color corresponds to the incidence.

A total of 218 cases of bites by non-native reptiles were recorded in France between 1 January 2000 and 31 December 2020. This represented 10.4 cases per year; however, the number of cases reached a maximum of 18 in 2016 (Figure 1). This represented an incidence of 3.8 per one thousand cases involving an animal (min. 0.5; max. 6.9) or 5.9/100,000 cases managed yearly by the PCCs (min. 0.9; max. 9.2). The bites were reported in all French regions, as described in Figure 2. 

### 2.1. Sociodemographics

One hundred and forty (64.2%) of them were male, compared with 78 (35.8%) females. The mean age of the patients was 29.0 ± 15.8 years (ranging from 4 months to 67 years). For seven patients, the age was unknown. Sixteen (7.3%) were younger than 6 years; 15 (6.9%) were aged 6–12 years; 19 (8.7%) were aged 13–18 years; and 161 (73.9%) were older than 18 years. The mean age of the patients bitten by a venomous species was higher than those bitten by a reputedly non-venomous species (39.5 ± 16.6 years vs. 26.7 ± 13.7 years, *p* < 0.001) (Table 1).

### 2.2. Circumstances of the Exposure

The number of reported bite cases per year has increased over the whole period. The time of the bites is described in Table 1 alongside the context (private or occupational). The cases of occupational bites involved the following situations: one involved a professional breeder; and another, a vet who was nursing a *Bitis nasicornis* at home. Seven cases occurred in pet shops, two in a breeding farm, and ten in a vivarium; all of these involved professionals selling or breeding reptiles. Finally, four cases occurred in the public during handling in a commercial context (fair, circus, photography studio, street show). The context was unknown in seven cases.

Only six patients admitted alcohol consumption at the time of the bite. One case of a bite by a venomous animal occurred in a patient who was drunk and high on drugs.

The current activity at the time of the bite was known in seventy-five cases (34.4%), as reported in Table 1.

### 2.3. Species Involved

The number of different species involved each year appears to have increased overall over the study period, with a maximum of 18 in 2016. All of the species involved are reported in Table 2.

### 2.4. Clinical Aspects

The bites occurred on the upper limb in 190 cases (87.2%) and on the lower limb in two cases. In one case, there were multiple bites on both the upper and lower limbs. There were nine bites to the head, almost exclusively by snakes belonging to the Boidae family (boids, 7 cases, including a 4-month-old child); however, also by a *Pantherophis guttatus* in a child, and a bite to the neck by a “rattlesnake” (unidentified species). Finally, there was one case of ocular projection of venom by a spitting cobra, *Naja mossambica*. 

The bite of *Heloderma suspectum* caused only localized symptoms (edema, pain), accompanied by vomiting and diarrhea. 

The bites by boids and pythonids were almost all of low severity (PSS 0/1) and associated with local edema, local pain, and even a hematoma or paresthesia. In one case of moderate severity (PSS 2), a patient presented with an edema of the whole hand; this was associated with paresthesia, local pain, and functional impotence of four fingers; and another had a local cutaneous infection.

The patient bitten on the thumb by *Thrasops flavigularis* experienced a burning and crushing sensation, followed by an edema that progressed to the forearm with localized pain along the lymphatic pathway. A diffuse hematoma along the arm was also present. Conversely, bites by other colubrids *(Lampropeltis* spp., *Pantherophis* spp., and *Elaphe schrenckii*) were consistently mild. In two patients, dysesthesia was also observed. The link between a bite and an infectious syndrome was questioned in two cases; however, no differential diagnosis was evoked.

The bite of *Ramphiophis oxyrhynchus* had no consequences other than a local cutaneous edema. On the other hand, in 3/7 cases of bites by *Heterodon nasicus*, the initial edema was accompanied by paresthesias of the bitten limb. Finally, in one case, the evolution was not favorable; at hour 12 of the bite, a phlegmon of the digitalocarpal sheath of the fifth finger was observed, associated with a septic and necrotic wound at the bite site.

The bite by *Aspidelaps lumbricus infuscates* was only a dry bite, resulting in no symptoms other than skin puncture. The ocular projection of venom by *Naja mossambica* resulted in keratitis without uveitis. Bites by the other elapids (including *Oxyuranus microlepidotus)* were all followed by a local syndrome (local edema up, localized external bleeding or hemorragic lymhedema, and local necrosis). The two cases of *Naja naja* bites resulted in a minimal neurotoxic syndrome.

The bites by *Viperinae* had only local consequences, with the notable exception of the one bite by *Proatheris superciliaris*. It led to a hematotoxic syndrome characterized by an edema extending to the groin and necrosis at the bite site. He rapidly developed an acute kidney injury due to renal artery thrombosis; this also explained the severe abdominal pain and, above all, a hypertensive crisis. A diagnosis of renal cortical necrosis, following disseminated intravascular coagulopathy, was finally made. The symptoms reported by patients bitten by crotalids are listed in Table 3. Among the North American rattlesnake bites, there was one case of a dry bite by a juvenile rattlesnake (undetermined species). The patient reported that the venom glands had been removed prior to purchase. 

The severity of the cases is summarized in Table 1. There was no reported death following a bite; however, two cases were left with sequelae, following amputation of a finger in one case of a bite by *Naja naja* and of a forearm following a bite by *Bothrops asper.*

### 2.5. Care Management

All of the patients bitten by a venomous reptile were hospitalized. Only twenty-eight cases were bitten by an animal that can be treated with an antivenom available in metropolitan France. Of the patients envenomed, only 10 (35.7%) were administered antivenom. They had been bitten by *Crotalus* sp., *Crotalus atrox*, *Crotalus polystictus*, *Crotalus durissus*, *Bothriopsis taeniata*, *Bothrops asper*, and received Antivipmyn Tri^®^ (Bioclon); by *Bothrops moojeni* and received Bothrofav^®^ (Sanofi); by *Naja naja* and *Naja annulifera*, and received FAV Afrique^®^ (Sanofi); and by *Bitis nasicornis* and (Inoserp Panafrica^®^). The other patients did not receive any antivenom for one of the following reasons: the grade of envenomation did not justify the use of antivenom (15 cases); the administration of antivenom was refused by the patients (four cases); a lack of knowledge of the species and/or the spectrum of action of the antivenoms (one case); and insufficient paraspecificity (two cases). 

Two patients underwent fasciotomy *(Crotalus atrox*, despite the administration of six vials of antivenom, and *Thrasops flavigularis).* There was also a case of surgical amputation of the forearm *(Bothrops asper)* and thumb *(Naja naja)* because of local necrosis.

## 3. Discussion

This work was a comprehensive retrospective study of bites by non-native reptiles reported in French PCCs. In France, bites by native vipers cause between 200 and 300 victims per year [5]. In 218 cases of bites by exotic reptiles in 20 years, i.e., an estimated incidence of 3.5 cases per one million inhabitants and less than 5% of the cases of envenomation by reptiles in France, the phenomenon might seem negligible. Indeed, in other countries, the number of bites by non-native snakes far exceeds that attributable to native species. For example, in Hungary, between 1970 and 2006, although the number of bites was comparatively lower (97 cases), 62.9% of them were caused by an exotic snake [6]. In the Czech Republic, 87 cases of bites by Viperidae or Elapidae were recorded in 15 years (1999–2013); or 0.06/100,000 inhabitants/year [7]. Finally, in an article published by four French and German PCC’s, 155 cases of bites by exotic snakes were reported between 1996 and 2006 [8]. 

The highest incidence of the reported bites was in the regions of Hauts-de-France, Grand-Est, and Provence-Alpes-Côte d’Azur. These are highly urbanized and border regions. According to a WWF report, some retailers’ premises are strategically located and easily accessible to the residents of neighboring countries. Large fairs in Germany or in the Netherlands attract large numbers of reptile enthusiasts [9].

The profile of the patients bitten is similar to what has already been reported from other countries [6,10]. The bite victim was a male patient under 30 years of age. The patients bitten by venomous animals were older; these animals were likely being kept by people with more experience in keeping reptiles in captivity. The presence of (sometimes very young) children in our study is evidence of the presence of animals in the home. Although rarely asked during telephone consultations or even admitted by patients, fatigue and the use of alcohol or drugs are factors that favor bites, as already reported [11]. The single case of envenomation at night by a rattlesnake suggests that the owners of such animals are more cautious.

In just over one in ten cases, the bite occurred in a professional context. The bites were significantly more serious in this case. Several breeding structures were represented in our study. Pet shops sometimes employ staff with little knowledge of the dangerousness of animals and safe care practices [12]. Conversely, the staff of farms for research or venom collection purposes, as well as vivaria open to the public, are better informed of the risks they run. All of them host potentially more dangerous animals than at home, either because of the specific need for certain venoms (for antivenom production) or to meet the expectations of visitors looking for thrills [12].

The activity at the time of the bite shows that certain situations are risky, namely nursing, feeding, and handling. These data are consistent with the results of previous studies in the United States and Australia [10,13]. Food excitement is probably involved in animals that are well fed, but all the more prone to inoculate venom as the possibility of escape is reduced [14]. Obviously, handling is all the more likely because the animals are considered to be non-venomous and not very aggressive. Several avoidable manipulations could have reduced the number of bite cases, in particular during demonstrations to the public, photography sessions, etc. Bites occur mainly on the upper limb (87.2% of cases), in contrast to bites by indigenous snakes [5,15]. Large animals, such as boas, which are thought to be placid, and which owners deliberately place around their necks, tend to bite at the head.

A total of twenty-three different species were identified, each with a highly variable number of bite cases. This is probably a consequence of the popularity of these animals. In Europe, the number of reptiles in households is estimated at 7.9 million, with France leading the way with 2.2 million animals, 1 million more than Germany, Italy or Spain [16]. As has been shown, the market for exotic animals is dominated by a small number of popular taxa, especially as the possibility of obtaining color variations is important [17]. Currently, the most successful reptiles in the snake category are *Pantherophis guttatus* and *Boa constrictor*. The latter is behaviorally reliable and adapts well to life in captivity. Thus, in the present study, we recorded numerous cases of bites by species which are the most frequently held at home.

The maxillary and mandibular glands of the powerful constrictor snakes, boa and python, are essentially mucous [18]. Nevertheless, the development of powerful teeth can lead to deep wounds and clinical manifestations; this is sometimes attributed to a toxic potential, but only due to the location of the bite or a disproportionate human reaction [19]. Moreover, due to their size, these snakes represent a potential danger for humans and especially for children, for whom a constriction can be fatal [20,21]. 

The same applies to colubrids which, although lacking Duvernoy’s glands or specialized dentition, were likely, in our study as in the literature, to cause a symptomatology that goes beyond simple cutaneous effraction. Although this is questionable for *Lampropeltis*, *Elaphe*, or *Panterophis*, the case of a bite by *Thrasops falvigularis* (the only one reported in the literature [22]) should raise caution. This animal belongs to a tribe that also includes other well-known dangerous species, such as *Dyspholidus typus* or *Thelotornis* spp. [23,24]. Close to the Colubridae, *Heterodon nasicus* (Dipsadidae) causes bites which, although not followed by signs of systemic envenomation, are responsible for moderate to severe local signs (edema, ecchymosis, blisters, and a burning sensation) [25]. Thus, for snake bites, there are clinical manifestations with marked local or even systemic signs in favor of a real envenomation with aglyphous snakes *(Heterodon*, *Thrasops)*. Some opisthoglyphous species with strong posterior maxillary teeth associated with Duvernoy’s glands could represent an increased risk of envenomation; however, these are very poorly illustrated in this series as they are rarely kept in captivity in France. The only case we report is the bite by *Rhamphiophis*, which did not have the severity that has already been described for this species [26].

The possibility of microbial infection following snakebites cannot be ruled out. Bacteria from the oral cavity of snakes such as Gram-negative enterobacteriaceae (e.g., *Morganella morganii*, *Proteus* spp.), can colonize wounds, and lead to local and systemic complications that can affect the patient’s prognosis [27,28]. Even relying on the kinetics of the onset of symptoms may not help given the sudden onset of some infections in animal bites (e.g., *Pasteurella*) [29].

Patients bitten by Elapidae or Viperidae presented neurotoxic or hematotoxic syndromes of varying intensity, respectively, and were consistent with the pictures already widely described ([30,31,32,33,34]). *Naja mossambica* is a species of African cobra whose ophthalmic effects of venom projection have been well described [35]. Kidney injury caused by the bite of *Proatheris superciliaris* is a serious, although usual, consequence of envenomation [36]. The low severity of bites by *Trimeresurus*, popular Asian crotalids because of their beautiful colors, is consistent with observations from the regions in which they are native [37,38]. 

In order to assist clinicians in the management of bites by venomous species, French breeding centers and PCCs joined forces in 2003 in an association called the ‘Banque des sérums antivenimeux (BSA)’ [39,40]; the aim of the association is to purchase foreign anti-venomous serum that meets French standards of safety and efficacy. The financing of the doses is ensured by the contributions of the breeders themselves. As antivenoms have the status of medicines, they cannot be held outside a hospital pharmacy. The antivenoms are thus distributed among four hospital sites in France, allowing the rapid delivery of the first vials required for treatment. The use of an antivenom has been the only effective specific treatment for snakebites for almost 120 years [41]. The mortality rate from this type of envenomation is extremely low in Western countries, even in the absence of antivenom, probably because of the available means of symptomatic resuscitation [42]. 

## 4. Limitations

This work presents the usual limitations of missing data in retrospective studies, for two different reasons. There is no standardized data collection specific to envenomations. Thus, biological results may be missing; especially if they are normal and circumstantial data are too infrequently collected, particularly by toxicologists that are not accustomed to these kinds of cases. Furthermore, despite efforts to complete the data, the questioning of patients is not always conclusive. For example, few patients admit to using drugs or alcohol. Similarly, patients might be unfamiliar with the species they hold, especially when it is acquired through illegal channels or is the result of a crossbreed for aesthetic purposes.

## 5. Conclusions

Most reported venomous reptile encounters involved animals kept in private collections and handled by men. Most bites involved very popular groups of non-venomous reptiles, such as pythons, boas, and colubrids. The most frequent venomous snakes were crotalids from Asia and the Americas, followed by African elapids. A third of venomous snakebites lead to antivenom use due to the limited availability of a specific antivenom, mild symptom severity, or patient refusal. 

## 6. Materials and Methods

A retrospective observational study was conducted in all the reported cases of snake bites from 2000 to 2020 in French PCCs. The cases were extracted from the PCC information system, which compiles all the exposure cases collected by the French PCCs during their telephone response to toxicological emergencies.

### 6.1. Selection of Cases

All the cases of bites by a reptile animal belonging to the snakes, anguimorphs (varanids), and iguanids groups, not native to mainland France and reported to the French PCCs, were included. 

The following data were analyzed: socio-demographic aspects (age, sex, geographical region of the case, place, and time of the bite); the species of reptile involved; the location of the bite; clinical manifestations following the bite; and the severity of the bite. Only the proteoglyphic and solenoglyphic snakes (Elapidae and Viperidae), and the lizard genus *Heloderma*, were considered venomous in the analysis of the cases.

Severity is calculated from the symptoms, the results of paraclinical examinations, and some recorded medical management. We used the poisoning severity score (PSS) [4]. The severity has five levels: PSS 0, no symptoms; PSS 1, mild severity; PSS 2, moderate severity; PSS 3, high severity; and PSS 4: death.

The treatments used, when they involved the use of antivenom, were also analyzed. Additional information was sought in the records: the patient’s activity at the time of the bite; the professional or extra-professional context of the bite; and the concomitant use of substances (alcohol, drugs) that could impair judgment or reflexes.

### 6.2. Statistics

We used R software to compare the qualitative variables using the Chi-squared and Fisher exact tests.

## Figures and Tables

**Table 1:** Distribution of exotic reptile bites.

Variable	Total	PSS * 0/1	PSS * 2/3	*p*
**Age (y.o.)**	211	27.5 ± 15.8	37.8 ± 13.3	<0.001
**Sex**				0.039
Male	140	115	25	
Female	78	72	6	
**Venomous animal**				<0.001
Yes	42	18	24	
No	168	161	7	
**Time of bite**				N.S.
Day	120	101	19	
Evening	72	62	10	
Deep night	23	21	2	
**Activity**				N.S.
Feeding, nursing	41	31	10	
Handling	34	31	3	
**Context**				<0.01
Private	187	166	21	
Occupational	24	15	9	
**Location**				0.022
At home	186	164	22	
At work	20	13	7	
Pet shop, fair…	5	5	0	

* PSS: Poisoning severity score [4]. See Section 6.

**Table 2:** Species involved.

Species	No. of Bites *n* = 218	PSS * 0 *n* = 98	PSS 1 *n* = 89	PSS 2 *n* = 23	PSS 3 *n* = 8
**Lizards**	7	4	2	1	
“Exotic lizard”	1		1		
* Pogona vitticeps*	1	1			
* Heloderma suspectum*	1			1	
*Varanus* sp. (incl. *V. exanthematicus*)	3	1	1		
* Iguana iguana*	1	1			
**Snakes**	211	94	87	22	8
“Exotic snake”	1	1			
“Snake from Guyana”	1		1		
**Elapidae**	8	1	2	4	1
* Aspidelaps lumbricus infuscates*	1	1			
“African naja”	1		1		
* Naja mossambica*	1			1	
* Naja annulifera*	1			1	
* Naja atra*	1		1		
* Naja naja*	2			1	1
* Oxyuranus microlepidotus*	1			1	
**Viperidae: Viperinae**	5		1	3	1
* Bitis nasicornis*	1			1	
* Cerastes vipera*	1		1		
* Cerastes cerastes*	1			1	
* Daboia palestinae*	1			1	
* Proatheris supercialiaris*	1				1
**Viperidae: Crotalinae**	28	4	10	9	
* Agkistrodon contortrix*	2		1	1	
* Bothriechis schlegelii*	1		1		
* Bothriopsis taeniata*	1				1
* Bothrops asper*	1				1
* Bothrops atrox*	1			1	
* Bothrops moojeni*	1	1			
* Crotalus sp.*	2		1	1	
* Crotalus atrox*	1				1
* Crotalus adamanteus*	1				1
* Crotalus durissus (incl. C. d. durissus and unicolor)*	3	1		1	1
* Crotalus polystictus*	1			1	
* Crotalus viridis oreganus*	1		1		
* Trimeresurus albolabris*	6	1	4	1	
* Trimeresurus flavomaculatus*	2		1	1	
* Trimeresurus schultzei*	1		1		
* Trimeresurus trigonocephalus*	2	1		1	
* Trimeresurus venustus*	1			1	
**Pythonidae**	69	37	3	1	
* Malayophython reticulatus*	1		1		
*Morelia* sp.	2		2		
*Morelia spilota* (incl. *M. s. cheyeni* and *M. s. macdowelli*)	2		2		
* Morelia viridis*	1	1			
*Python* sp.	26	16	10		
* Python molurus*	5	3	2		
* Python regius*	32	17	14	1	
**Boidae**	43	24	18	1	
*Boa* sp.	20	12	8		
* Boa constrictor*	18	9	8	1	
* Boa imperator*	4	2	2		
* Eryx colubrinus*	1	1			
**Colubridae**	48	27	18	3	
“Exotic colubrid”	3	1	2		
* Elaphe schrenckii*	1	1			
*Lampropeltis* sp.	2	1	1		
* Lampropeltis californiae*	4	3	1		
*Lampropeltis triangulum* (incl. *L. t. hondurensis* and *L. t. campbelli*)	4	2	2		
*Pantherophis* sp.	1	1			
* Pantherophis bairdi*	2	1	1		
* Pantherophis guttatus*	29	17	10	2	
* Pantherophis obsoletus*	1		1		
* Thrasops flavigularis*	1			1	
**Dipsadidae**	6		5	1	1
* Heterodon nasicus*	7		5	1	1
**Lamprophiidae**	1		1		
* Rhamphiophis oxyrhynchus*	1		1		

* PSS: Poisoning severity score [4]. See ‘Material and methods’ section.

**Table 3:** Reported signs, symptoms and biological disturbances in the patients bitten by crotalids.

Reported Signs or Symptoms		Number of Cases
Local signs	Erythema	12
	Pain	11
	Edema	10
	Necrosis, blisters	4
	Ecchymosis	4
	Bleeding at the skin puncture	1
	Compartment syndrome	1
Systemic signs	Extensive edema	3
	Adenopathy	3
	Tachycardia	2
	High blood pressure	2
	Paresthesia	1
	Organic acute kidney injury	1
	Hyperthermia	1
	Low blood pressure	1
	Extensive ischemia	1
Biological perturbations	Coagulopathy	3
	Increased prothrombin time	3
	Hyperleukocytosis	3
	Thrombocytopenia	2
	Rise in CK	2
	Hypofibrinogenemia	1

## Data Availability

Not applicable.
